# Review: The Application of MXene in Thermal Energy Storage Materials for Efficient Solar Energy Utilization

**DOI:** 10.3390/ma18122839

**Published:** 2025-06-16

**Authors:** Han Sun, Yingai Jin, Firoz Alam

**Affiliations:** 1 National Key Laboratory of Automotive Chassis Integration and Bionics, Jilin University, Changchun 130022, China; sunhan24@mails.jlu.edu.cn; 2 College of Automotive Engineering, Jilin University, Changchun 130022, China; 3 School of Engineering (Aerospace, Mechanical and Manufacturing), RMIT University, Melbourne, VIC 3000, Australia; firoz.alam@rmit.edu.au

**Keywords:** MXene, photothermal conversion, thermal storage, phase change materials

## Abstract

Two-dimensional transition metal carbides/nitrides (MXenes) have shown potential in biosensors, cancer theranostics, microbiology, electromagnetic interference shielding, photothermal conversion, and thermal energy storage due to their unique electronic structure, ability to absorb a wide range of light, and tunable surface chemistry. In spite of the growing interest in MXenes, there are relatively few studies on their applications in phase-change materials for enhancing thermal conductivity and weak photo-responsiveness between 0 °C and 150 °C. Thus, this study aims to provide a current overview of recent developments, to examine how MXenes are made, and to outline the combined effects of different processes that can convert light into heat. This study illustrates the mechanisms that include enhanced broadband photon harvesting through localized surface plasmon resonance, electron–phonon coupling-mediated nonradiative relaxation, and interlayer phonon transport that optimizes thermal diffusion pathways. This study emphasizes that MXene-engineered 3D thermal networks can greatly improve energy storage and heat conversion, solving important problems with phase-change materials (PCMs), like poor heat conductivity and low responsiveness to light. This study also highlights the real-world issues of making MXene-based materials on a large scale, and suggests future research directions for using them in smart thermal management systems and solar thermal grid technologies.

## 1. Introduction

The Sun is the primary source of energy reaching the Earth’s surface, but its availability fluctuates with the time of day and weather conditions. In practice, this means that the energy received during the day is often insufficient to meet demand at night or during periods of low ambient temperature. As a result, the effective use of solar energy requires the ability to store thermal energy for later use. To address this challenge, various thermal energy storage (TES) systems have been developed to capture excess heat and release it when needed [1].

Phase-change materials (PCMs) have emerged as promising candidates for solar energy utilization [2], industrial waste heat recovery, and building efficiency applications, owing to their energy storage density and near-isothermal operation characteristics [3]. Nevertheless, conventional PCMs suffer from intrinsic limitations: thermal conductivities typically below 1 W/(m·K) and negligible photothermal conversion capabilities, which substantially hinder their synergistic integration with solar energy systems.

The photon-to-thermal energy conversion process, ubiquitous across physical, chemical, and biological systems, constitutes one of nature’s most fundamental energy transduction mechanisms. When materials function as photon absorbers capable of efficient light-to-heat transformation, this photothermal conversion phenomenon—recognized as the photothermal effect—represents one of humanity’s earliest approaches [4]. The photothermal performance of light-absorbing materials is principally governed by two intrinsic characteristics: photon harvesting capacity and energy conversion efficiency. Photon harvesting, the process whereby materials absorb and utilize incident radiation through physical/chemical mechanisms, serves as the critical initial phase in photothermal conversion, photocatalysis, and photovoltaic applications. This capability is intrinsically determined by complex interplays between microstructural configurations, electronic properties, and photon–matter interaction dynamics [5].

Contemporary research has identified three distinct photothermal conversion mechanisms categorized by material systems: (a) localized surface plasmon resonance (LSPR) effects in metallic nanostructures under illumination; (b) non-plasmonic interbond/intrabond electronic transitions in semiconductor materials; and (c) lattice vibration-mediated energy conversion in carbon-based materials and selected organic polymers [6]. The development of broadband-absorbing photothermal materials shows promise for enhancing renewable energy utilization efficiency. Concurrently, advanced heat-generating materials bear significant implications across diverse applications including desalination technologies [7], thermal energy storage systems [8], photothermal catalysis [9], and biomedical engineering [10]. However, conventional photothermal materials (e.g., metal nanoparticles, carbon-based substances) face inherent limitations, such as narrow absorption bandwidths, prohibitive costs, and complex synthesis protocols.

In this context, two-dimensional transition metal carbides/nitrides (MXenes) have emerged as a focal point in photothermal research, due to their good combination of tunable electronic structures, surface chemistry modulability, and higher photothermal conversion capabilities [11]. Table 1 highlights the relative advantages and disadvantages of selected photothermal materials.

**Figure 1:**
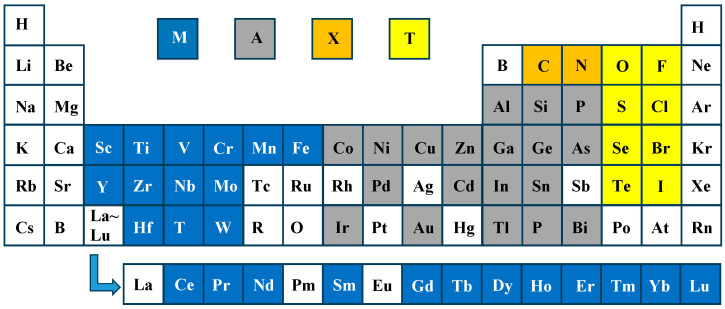
MXene and precursor (MAX) element composition.

MXenes, with the general chemical formula M_n+1_X_n_T_x_ (n = 1–4), are a class of two-dimensional materials where M represents early transition metals, X denotes carbon or nitrogen, and Tx signifies surface functional groups such as -O, -OH, or halogen atoms [15]. MXene and precursor element composition are shown in Figure 1. The study by Naguib et al. [16] demonstrated the synthesis of MXenes through the selective etching of aluminum layers from MAX phase precursors (Ti_3_AlC_2_) using hydrofluoric acid solution, which enabled the separation of tightly packed MX layers into individual 2D nanosheets. Recently, researches have experimentally confirmed over thirty (30) MXene variants, while theoretical calculations predict substantially more possible compositions [17]. Notably, twenty eight (28) nitride MAX phases and seventeen (17) nitride MXene configurations have been identified as thermodynamically stable, though many of these remain theoretical due to synthesis challenges [18]. The highly tunable metal element composition endows MXene with excellent mechanical strength and magnetic properties, and its conductivity is close to that of graphene [19]. The atomic spacing in the MXene lattice can be precisely tuned by modulating the surface functional groups, thus triggering abrupt changes in properties (e.g., superconducting properties) that are dependent on the surface functional groups [20]. VahidMohammadi et al. [21] suggested that MXene without surface groups is no different from a metallic conductor in terms of its electronic properties. Based on the calculations of density-functional theory (DFT), Feng et al. [22] suggested that Mo-MXene with OH- and F- as end groups has semiconductor-like properties, while O- exhibits metallic behavior when used as an end group. Since the carrier migration and thermal diffusion of MXene are confined to a two-dimensional plane, coupled with the oxidation/reduction activity of the surface transition metal atoms, MXene exhibits unique photothermal properties, photoelectrical/electrochemical properties, and catalytic capabilities [23].

These characteristics and features position MXenes as critical functional materials for applications in biomedical devices [24], water purification [25], supercapacitors [26], and sensor technologies [27]. The ongoing development of surface engineering techniques (e.g., the tuning of surface end-group ratios [28], construction of heterojunctions [29], etc.) continues to expand their performance envelope across multiple disciplines.

While MXene’s inherent metallic conductivity (9880 S·cm^−1^) [30] and exceptional theoretical capacitance (1982 F·cm^−3^) [31] have driven extensive exploration in electrochemical applications [32], its thermal energy storage potential in PCMs remains underexplored and least studied.

**Figure 2:**
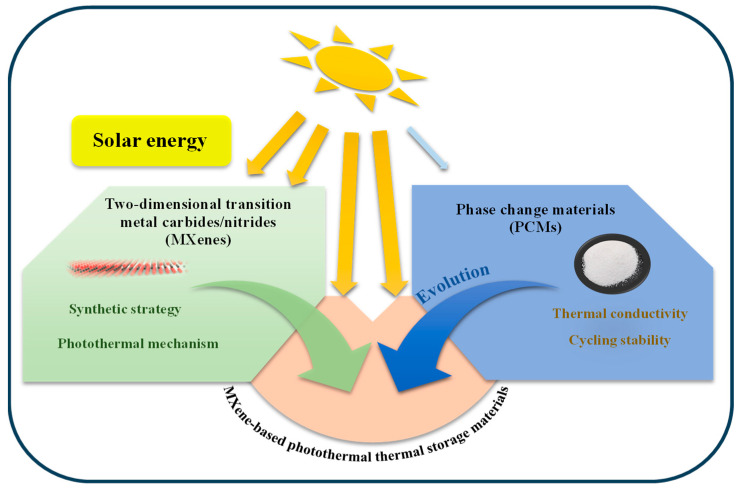
Overview of the solar-energy-assisted interplay between MXenes and PCMs.

Therefore, this study aims to carefully examine the latest research on MXene-based thermal storage materials, especially PCMs, by systematically analyzing recent advancements in MXene-enhanced thermal composites. Apart from background, introduction, and current need identification, the remainder of this article is organized in the following sequences: Section 2 specifies the principles to be followed for the preparation of MXene, as well as the commonly used preparation methods. Section 3 analyzes the physical mechanism of its photothermal conversion. Section 4 summarizes the construction strategies and performance enhancement principles of composite thermal storage systems based on current research, focusing on the constitutive relationship between microstructure/photothermal performance/thermal storage behavior of materials. This concise yet comprehensive perspective aims to redirect research focus toward MXene’s thermal management capabilities while providing a scientific roadmap for developing next-generation thermal energy storage systems. By correlating atomic-scale structural features with macroscopic performance metrics, this study establishes foundational principles for engineering high-efficiency, solar-responsive thermal storage materials, especially PCMs. The synergistic interplay of MXenes and PCMs, utilizing solar energy to advance the development of MXene-based photothermal energy storage materials proposed in this study, is illustrated in Figure 2.

Section 5 covers the industrial implications and provides a general overview of MXene-based PCMS in practical applications and problems. Section 6 concludes by outlining the key findings and suggestions for future research.

## 2. Preparation of MXene Materials

**Figure 3:**
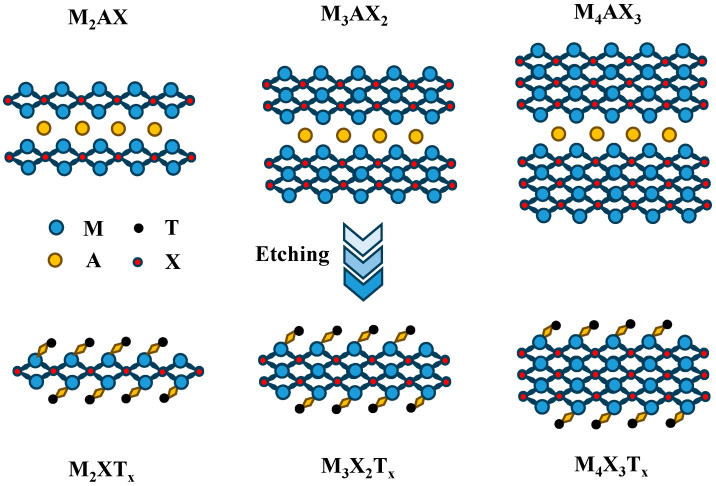
Influence of precursor MAX phase parameters on the structure of MXene.

MXenes are predominantly synthesized through top–down approaches [33], where the processing parameters of precursor MAX phases critically determine the final material’s characteristics, as shown in Figure 3. MAX phases are a family of ternary carbide or nitride ceramics all belonging to the hexagonal crystal system and having a layered structure. Their general formula is M_n+1_AX_n_, where n usually equals 1~3, M is a transition metal element, A is an A group element, and X is C or N [34]. The inherent bonding configuration in MAX phases—strong intra-layer ionic/covalent M-X bonds combined with weaker interlayer metallic M-A bonds—fundamentally governs their exfoliation behavior [35]. This chemical asymmetry enables the selective removal of A-layer atoms through acidic etching solutions or molten salt treatments while preserving the MX backbone. The material’s exfoliability can be quantitatively predicted by calculating the maximum exfoliation energy, which correlates with the relative strength difference between M-X and M-A bonds. Practical synthesis challenges emerge when M-A and M-X bond strengths approach parity, leading to uncontrolled phase dissolution rather than controlled layer separation [15]. In simple terms, the success of the stripping lies in whether the oxidation of the A layer and its products can be detached from the precursor smoothly during the etching process. In addition, the stripping energy is also related to the chemical properties of X. The presence of extra valence electrons in N compared to C results in a weaker M-N bond, thus affecting the stability of the nitride MXene to some extent [36]. Suitable exfoliation strength is the key to the preparation of MXene. This section specifically introduces the MXene synthesis methods, and summarizes the advantages and disadvantages of different synthesis strategies.

### 2.1. Fluorine-Containing Compound Etching

Since Naguib used hydrofluoric acid (HF) solution etching to create MXene in 2011, acid etching with fluoride has become the primary method for making MXene [37]. As mentioned earlier, the key step in the synthesis of MXene is the selective removal of the ‘A’ layer from the precursor, and the reaction principle when Ti_3_AlC_2_ is immersed in HF solution is shown in Equation (1).

(1) $${\mathrm{Ti}}_{3}{\mathrm{AlC}}_{2}+3\mathrm{HF}={\mathrm{AlF}}_{3}+\frac{3}{2}H_{2}+{\mathrm{Ti}}_{3}C_{2},$$

**Figure 4:**
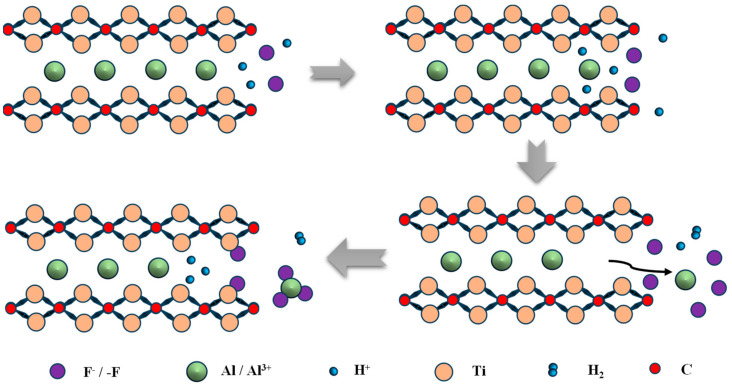
Etching process diagram (Ti_2_AlC).

During the entire MAX phase etching process, H^+^ ions facilitate the oxidation of aluminum layers. To prevent oxidized aluminum byproducts from hindering further H^+^ penetration into inner Al layers, these products must be converted into soluble species and removed from the precursor matrix—a process enabled by fluoride ions (F^−^) acting as ligands for Al^3+^. This mechanism typically yields MXenes with characteristic accordion-like morphology, resulting from hydrogen gas generation during etching; a similar process is schematized as shown in Figure 4 [38]. These observations confirm that effective etchants must simultaneously achieve both oxidation and removal of the A-phase.

In the etched Ti_3_C_2_ MXene structure, each formula unit exposes two titanium atoms capable of bonding with surface terminations like -F and -OH groups [16]. Hydrofluoric acid (HF) etching remains the preferred industrial-scale method due to its high yield and completeness of layer separation. Alhabeb et al. [39] established a standardized stepwise protocol for Ti_3_C_2_ synthesis via HF etching in 2017, though the method’s practical challenges—particularly the need to balance corrosive HF concentrations with etching duration—demand strict safety protocols. By contrast, Ghidiu et al. [40] developed a safer in situ etching approach in 2014 using fluorinated salt (e.g., LiF [41], FeF_3_ and NH_4_F [42]) and HCl mixtures. This method achieves comparable yields to HF etching (within experimental error margins) while enabling lithium–ion intercalation during processing, producing MXenes with distinctive clay-like textures. Although limited to specific MXene compositions requiring moderate etching intensity, this technique offers better control over product dimensions and quality, representing a significant advancement in precision synthesis [43].

### 2.2. Fluorine-Free Preparation

The continuous expansion of MXene variants has driven a transformative evolution in synthesis methodologies, with emerging fluoride-free synthesis protocols addressing both the environmental concerns and the performance limitations inherent in traditional approaches [44]. This paradigm shift toward environmentally conscious manufacturing eliminates hazardous fluorine compounds while simultaneously enhancing electrochemical activity through the avoidance of electrochemically inactive fluorine terminations. Therefore, the etching method of MXene has gradually evolved from the initial fluorine-containing etching method to the fluorine-free direction [45]. When utilizing Al-containing MAX phases as precursors, the amphoteric nature of aluminum theoretically permits hydroxide ions (OH^−^) to substitute fluoride as coordinating ligands. However, a practical implementation reveals that the intermediate oxide/hydroxide byproducts inevitably form passivation layers that inhibit complete Al removal [46]. Li et al. [47] reported that MXene with a mass fraction of 92% could be obtained by the hydrothermal treatment of Ti_3_AlC_2_ in a concentrated NaOH solution with a specific capacitance of 314 F·g^−1^. This surpasses (by ~214%) that of the MXene prepared via the conventional HF-based etching route, which typically yields -O and -OH as the predominant end groups and requires strict control over hydrothermal temperature and NaOH concentration. The removal of Al can also be realized electrochemically [48], and the customized electrolyte can selectively remove Al and replace it with -O and -OH, as follows in Equation (2) [49]:

(2) $${{\mathrm{Ti}}_{3}{\mathrm{AlC}}_{2}\;-\;3e}^{-}{={\mathrm{Ti}}_{3}C_{2}+\mathrm{Al}}^{3+},$$

This method is capable of extracting MXene with sizes up to 18.6 mm MXene flakes, and the yields were all over 90%.

The synergistic combination of anodic aluminum layer oxidation and cathodically controlled proton insertion enables synchronized Al^3+^ dissolution with concurrent surface functionalization. For non-aluminum MAX phases like Ti_3_SiC_2_, the reduced atomic radius creates stronger Ti–Si bonding that necessitates supplemental oxidizers (e.g., HNO_3_ or H_2_O_2_) to facilitate etching [50]. Lewis acidic molten salt etching has emerged as a versatile alternative, particularly for A-site elements like Zn, Si, or Ga, operating through a dual mechanism as follows: (i) high-temperature oxidation of A-layer atoms by metallic cations in the molten salt, accompanied by anion-driven surface termination formation, and (ii) reductive precipitation of excess cations onto MXene surfaces as metallic deposits subsequently removable through solution washing [51]. While this strategy enables precise control over surface terminations dictated by the molten salt’s anion composition, and circumvents interlayer expansion issues caused by hydrophilic -O/-OH groups, the resulting MXenes predominantly remain in multilayered configurations with limited delamination efficiency [52].

Intercalation-assisted delamination has emerged as a versatile strategy for isolating single-layer MXene sheets, where the insertion of chemical species, like dimethyl sulfoxide (DMSO), between the MXene layers followed by aqueous ultrasonication produces stable colloidal suspensions that can be filtered to yield high-quality monolayers [53]. MXene can also be prepared by chemical vapor deposition (CVD) [54], which provides high crystallinity and low defects, which is beneficial for the study of material properties. However, the synthesis process is complicated and less efficient, making it difficult to achieve large-scale commercial production.

### 2.3. Degradation Issues of MXene

**Figure 5:**
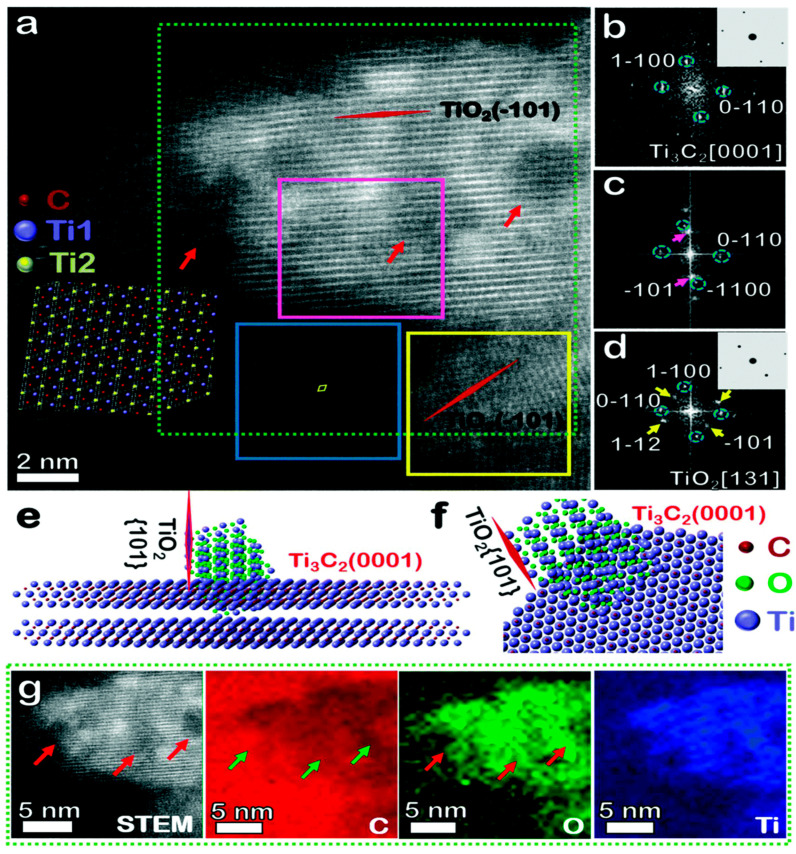
Orientation relationship between anatase TiO_2_ and Ti_3_C_2_ MXene. (**a**) A Z-contrast STEM image is showing the formation of two TiO_2_ nano- particles on the substrate of Ti_3_C_2_ MXene; (**b**–**d**) FFT patterns of the areas are marked by blue, pink, and yellow boxes in (**a**), respectively, and their comparison with simulated diffractions (inset) assuming an anatase structure; (**e**,**f**) are illustrations of the TiO_2_ cluster on Ti_3_C_2_ with the TiO_2_-[101] plane perpendicular to the MXene basal plane [0001], while (**g**) displays elemental maps of C, O, and Ti collected by EELS, showing the TiO_2_ cluster with a clear lattice and amorphous carbon aggregation nearby [57].

MXene’s environmental stability presents a critical challenge for practical applications, as ambient oxygen and moisture induce progressive degradation that alters its composition, structural integrity, and surface functionality [55]—ultimately compromising material performance and lifespan [56]. Advanced characterization techniques, including scanning transmission electron microscopy (STEM) and electron energy loss spectroscopy (EELS), have revealed oxidation mechanisms where titanium atoms preferentially form TiO_2_ crystallites at the sheet edges and basal planes, while residual carbon aggregates into amorphous domains [57], as shown in Figure 5. This degradation pathway originates from inherent atomic defects created during synthesis, which Cao et al. [58] identified as primary nucleation sites for oxidation initiation.

Degradation kinetics are dramatically accelerated by simultaneous hydrolysis reactions in an aqueous environment under room temperature conditions (23 °C). Experimental studies have revealed complete MXene decomposition within 15 days of ambient aqueous exposure, with a sequential transformation observed for 42% degradation at 5 days progressing to 85% at 10 days, ultimately yielding turbid colloidal solutions containing TiO_2_ nanoparticles [59]. To address these stability limitations, Maleski et al. [60] compiled a solvent library for MXene dispersion, identifying organic media like N-methyl-2-pyrrolidone (NMP) and dimethyl sulfoxide (DMSO) that suppress oxidation through surface passivation and oxygen scavenging. These optimized storage protocols can extend MXene shelf-life from hours to months while maintaining >90% material integrity.

Environmental degradation mitigation strategies for MXene-based materials center on advanced encapsulation technologies, where moisture-blocking polymers and gas-impermeable barrier films create protective interfaces that prevent oxidative species penetration during storage and operational phases [61]. These evolving protection protocols are being tailored for specific application requirements—from flexible electronics needing mechanical durability to energy storage systems demanding long-term electrochemical stability—marking a critical step toward practical MXene implementation across diverse technological platforms.

## 3. MXene Photothermal Conversion Mechanism

**Figure 6:**
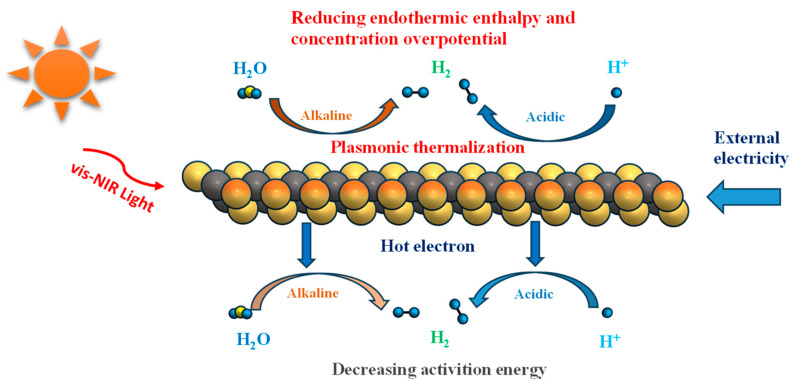
Schematic illustration of the LSPR-induced photothermal and hot-electron effect that can improve the electrocatalytic HER performance of MXenes.

MXene’s photothermal conversion arises from synergistic multiscale mechanisms operating through three sequential phases: photon absorption, energy transformation, and thermal dissipation. At subwavelength dimensions, MXenes exhibit LSPR effects—a phenomenon where conductive electrons undergo collective oscillations when interacting with incident light [62]. These plasmonic nanostructures overcome classical diffraction limits by confining electromagnetic fields to nanoscale volumes, dramatically enhancing light–matter interactions. The quantized energy states of these electron oscillations, termed plasmons, generate two critical effects, including intense near-field electromagnetic enhancement and resonant-frequency absorption/scattering cross-section amplification, establishing MXenes as exceptional photon harvesting platforms [6]. Building upon these fundamentals, MXenes show better plasmonic performance through their intrinsic high carrier density (8 ± 3 × 10^21^ cm^−3^ in monolayer Ti_3_C_2_T_x_) and broadband spectral absorption spanning visible to near-infrared regions (vis-NIR), as shown in Figure 6 [63]. The spectral absorption range is also related to the composition and structure of MXene. Maleski et al. [64] first quantitatively listed the spectral properties of M_n+1_C_n_ MXene when M and n are varied. Among other things, a decrease in the value of n (e.g., Ti_3_C_2_ and Ti_2_C) leads to a shift of the main excitation peaks in the spectra to higher energies, and a complete or partial replacement of Ti with Mo leads to the same result.

Surface termination further modulates absorption profiles, with different MXene compositions exhibiting tunable plasmon peaks across the vis-NIR spectrum [21]. Upon photon absorption, covalent bond excitation generates high-energy electron-hole pairs that undergo rapid non-radiative relaxation—a process where kinetic energy transfers to lattice phonons through electron collisions rather than photon emission. MXene’s metallic conductivity facilitates ultrafast electron transport, minimizing charge recombination while maximizing thermal conversion efficiency [65]. Density functional theory simulations (DFT) by Zhang et al. [66] reveal that photoexcited electrons in Ti_3_C_2_ preferentially transfer energy to TiO_2_-derived phonon modes, creating localized hotspots that amplify thermal output. This unique energy cascade mechanism positions MXenes as quintessential photon-to-heat converters with engineered thermal dissipation pathways.

## 4. MXene-Based Composite Heat Storage Materials

Thermal energy storage systems have progressively been becoming indispensable across diverse large-scale applications, including power generation plants, geothermal installations, nuclear facilities, smart textiles, architectural thermal regulation, food processing infrastructure, and solar energy harvesting/storage technologies [67]. As the functional core of these systems, PCMs exhibit exceptional heat storage/release capacities through phase transitions. While substantial advancements have expanded PCM varieties, critical limitations persist—particularly low thermal conductivity (typically 0.1–0.5 W/(m·K)), pronounced overcooling hysteresis, and cycling instability that constrain practical implementation [68]. This section analyzes the breakthrough studies demonstrating MXene’s transformative potential in PCM enhancement.

### 4.1. Improvement of Heat Storage Capacity

Thermal conductivity stands as a critical determinant of phase change materials’ (PCMs) energy storage performance [69]. Insufficient thermal conductivity not only prolongs heat transfer durations but creates temperature gradients within PCM matrices, potentially triggering phase separation due to delayed thermal equilibration [70]. Organic PCMs, such as paraffin, suffer from inefficient phonon-mediated heat transfer caused by weak van der Waals interactions between loosely packed molecules. While inorganic counterparts, such as hydrated salts, possess ordered crystalline structures, inherent limitations—including grain boundary scattering, lattice defects, and phase-change-induced volume expansion—severely impede thermal transport through intensified phonon dissipation [71].

Conventional thermal enhancement strategies employing conductive additives often backfire through interfacial thermal resistance at filler-PCM boundaries, where excessive additive loading paradoxically reduces energy storage density through compromised heat transfer efficiency [72]. MXene-based composites fundamentally overcome these limitations through structural engineering. When integrated with PCMs, MXene’s layered structure provides ordered channels for molecular alignment, enabling tight interfacial bonding as PCM molecules occupy interlayer spaces and adhere to MXene surfaces [73]. This hierarchical architecture establishes continuous thermal pathways where interconnected MXene networks facilitate efficient phonon propagation, effectively reducing thermal charge/discharge durations compared to pure PCM systems [74]. Experimental validation by Sheng et al. [75] showed measurable improvements—pure PEG exhibited 0.25 W/(m·K) thermal conductivity versus 0.42 W/(m·K) for MXene/PEG composites. The moderate enhancement suggests incomplete 3D thermal network formation at low MXene loadings, highlighting the need for optimized dispersion techniques to fully exploit MXene’s anisotropic thermal transport capabilities.

Undercooling presents another critical challenge in PCMs’ thermal performance [76], where liquid persistence below the crystallization temperature delays heat release and disrupts phase transition cycles, ultimately degrading energy storage efficiency [77]. MXene composites effectively mitigate this limitation by leveraging their hierarchical porous architecture—the nanoscale cavities and surface defects provide abundant heterogeneous nucleation sites that promote additional crystallization initiation points [69]. This engineered nucleation mechanism significantly reduces undercooling hysteresis by lowering the activation energy barrier for solid-phase formation, ensuring synchronized phase transitions during thermal cycling.

**Figure 7:**
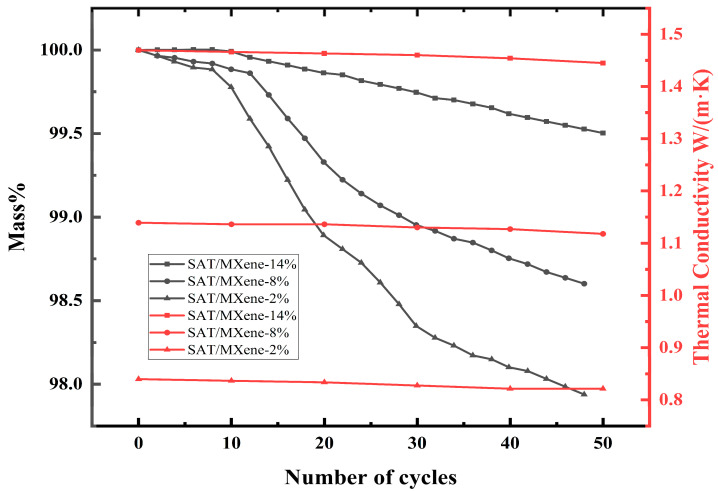
Thermal conductivity and mass variation curves of SAT/MXene CPCMs with the number of cycles.

Cycling stability serves as a critical performance metric determining the long-term operational reliability of PCMs, where conventional systems suffer from progressive heat storage degradation due to material leakage during repeated thermal cycling [78]. MXene-based encapsulation strategies fundamentally address this limitation through dual mechanisms: the material’s ultrahigh specific surface area provides structural confinement, while capillary forces and interfacial tension within the MXene nanocomposites synergistically restrict molten PCM migration between the layers. The thermal conductivity and mass variation curves of SAT/MXene CPCMS as a function of cycles are illustrated in Figure 7 [73].

Du et al. [79] fabricated a biomass-derived phase change composite through erythritol impregnation into dopamine-modified MXene nanofibers. This phase change composite interconnected the porous architecture by physically stabilizing the erythritol matrix and chemically prevented liquid-phase leakage through surface interactions. The composite maintained consistent thermal charge/discharge profiles through 100 operational cycles, showing less than 3% enthalpy deviation—conclusive evidence of enhanced cycling stability surpassing conventional PCMs performance benchmarks.

### 4.2. Expansion of the Photothermal Capacity of PCMs

The evolving energy landscape demands have advanced PCMs that integrate thermal storage with solar energy harvesting capabilities. While traditional PCMs excel in heat management, their inherent inability to directly convert sunlight into storable thermal energy limits their applicability in renewable energy systems [80]. Solar energy, though abundant and cost-free at source, requires efficient capture technologies to maximize utilization—a challenge where current energy conversion methods (photovoltaics, wind/hydroelectric systems) incur efficiency losses through multi-stage energy transformations [81]. MXene-enhanced PCM composites present a paradigm-shifting solution by enabling direct solar-to-thermal energy storage [82]. This approach eliminates intermediate conversion steps through the development of light-triggered thermal storage-release systems, where MXene acts as both photon harvester and thermal conductor [83]. The material’s exceptional broad-spectrum absorption stems from synergistic plasmonic effects and surface functionalization. The -OH and -O groups enhance photon trapping through dipole interactions. Given the thin-layered nature of MXenes and their limited volumetric heat capacity, their contribution to thermal storage must be evaluated critically and may, at present, be more conceptual than practical. For photothermal conversion efficiency η, researchers have calculated efficiency using Equation (3):

(3) $$\eta =\frac{m\cdot \Delta H}{P\cdot S\cdot \Delta t}$$

where m denotes material mass, ΔH denotes enthalpy of melting, P denotes light intensity, S denotes light area, and Δt denotes melting time (light time).

**Figure 8:**
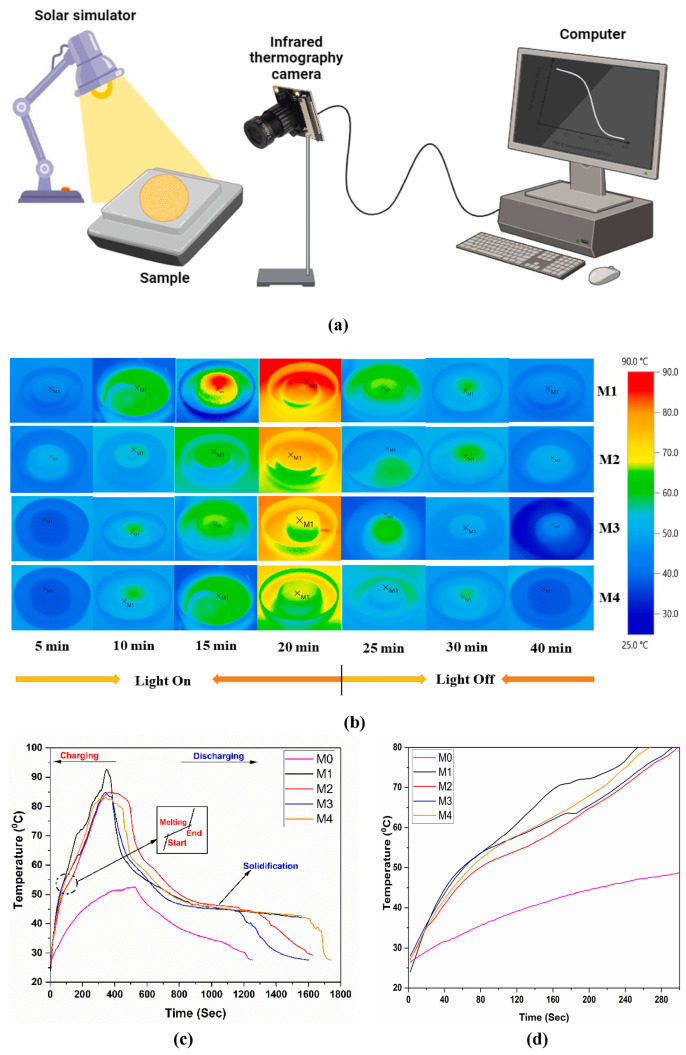
Schematic representation of (**a**) experimental setup for photothermal conversion, (**b**) infrared images of MXene enhanced eutectic PCMs, (**c**) temperature/time characteristics of MePCM sample for the charging/discharging cycle, and (**d**) detailed melting time in a desired interval [74].

Wang et al. [73] achieved an 81.3% photothermal conversion efficiency by integrating sodium acetate trihydrate (SAT) with MXene, expanding the material’s effective absorption spectrum from 200–258 nm to 200–497.7 nm. In a separate development, Panda et al. [74] obtained an MXene-modified eutectic PCM combining paraffin and polyethylene glycol (PEG). The photothermal experimental setup and the photothermal properties of the materials are shown in Figure 8. While the base eutectic mixture showed limited UV-range absorption (200–400 nm), the MXene composite exhibited broadband photon capture spanning 200–800 nm with dual absorption peaks. Under standardized illumination, the hybrid PCM reached peak temperature in 6 min—40% faster than the unmodified system—while achieving 98.53% conversion efficiency. Complementary research by Fan et al. [84] revealed that MXene’s localized surface plasmon resonance (LSPR) induces dual strong absorption bands in visible (450–650 nm) and near-infrared (750–900 nm) regions. This multiband photon harvesting mechanism, coupled with MXene’s anisotropic heat transfer pathways, enabled the composite to store 94.5% of incident solar energy as usable heat. These studies collectively validate MXene’s dual functionality as both broadband photon captor and thermal conductivity enhancer, establishing a new performance benchmark for solar-driven thermal storage systems. Table 2 in Section 4.2 shows more recent and relevant research work demonstrating the need for research on MXene composite heat storage materials. Notably, poly(ethylene glycol) (PEG) dominates the current MXene/PCM research due to its exceptional chemical compatibility with MXene surfaces—hydroxyl-rich PEG chains form hydrogen bonds with MXene’s oxygen terminations [85], achieving stable interfacial bonding crucial for long-term cyclability [86]. Kalidasan et al. [87] prepared MXene-based binary eutectic phase change materials with different mass fractions of sodium sulfate decahydrate/dodecahydrate MXene by a two-step method and focused on the photo-thermal cycling properties. The FTIR spectra of the composite PCM containing 0.9% MXene before and after undergoing 200 thermal cycles were similar, with each peak coinciding with the other at the same frequency, demonstrating good photothermal cycling performance.

## 5. Discussion and Industrial Implications

Despite the advantages demonstrated in enhancing PCM performance, MXene-enhanced composites face significant barriers to commercialization that demand systematic scientific resolution. Three critical challenges dominate current research limitations. These limitations are high cost, lack of comprehensive life cycle assessment, and multiscale modelling deficit. These shortcomings are elaborated as follows.

(a) Cost.

The synthesis pipeline—from MAX phase precursors to final MXene composites—remains cost-prohibitive due to toxic chemical requirements (e.g., HF etching) [101], energy-intensive processing, and precursor material costs. Although these components are already relatively inexpensive for Ti-based MXene, it is crucial to reduce the cost of precursors for other MXene (Ta, Hf, V) [102]. Ti_3_C_2_T_x_ was the first MXene to be discovered, but it is by no means the optimal MXene for every application, unless further research is carried out on a wider family of MXene. There is a significant discrepancy between the cost investment and the uncertain actual returns, and the lack of profit motivation is the key obstacle to commercialization.

(b) Life Cycle Assessment.

There is a lack of experimental data covering the complete lifecycle. Currently, most reported experimental data are obtained in instrumented environments. While these results facilitate the exploration of material composition and working mechanisms, they provide limited assistance in practical applications. The performance of composite PCMs, including mechanical properties, flame retardancy, moisture absorption, and the ease of secondary processing, needs further investigation in real-world scenarios. For the target working conditions of the material, equivalent application scenarios (e.g., building heating, wearable devices, etc.) should be established, and the experimental data (e.g., latent heat, thermal conductivity, and cyclic stability, etc.) and the potential toxicity safety issues (despite the better biocompatibility of MXene compared to other 2D materials, such safety issues are still related to differences in the composition and structure of the material [103]), among others, can demonstrate the material’s practicability.

(c) Multiscale Modeling Deficits.

The anisotropic nature of MXene’s layered architecture necessitates advanced multiscale modeling approaches currently underdeveloped. Bridging this gap requires the following:

- Quantum mechanical simulations of MXene/PCM interfacial bonding;
- Mesoscale finite element analysis of heat transfer pathways;
- Macroscopic system-level thermal performance modeling.

Emerging machine learning frameworks show promise in accelerating this multiscale integration, potentially reducing R&D cycles by 40–60%, while optimizing composite formulations through inverse design principles. Until such predictive capabilities mature, empirical trial-and-error approaches will continue dominating MXene/PCM development, perpetuating high costs and slow progress.

## 6. Conclusions and Prospects for the Future

MXene, which emerges as an innovative class of photothermal materials, provides a new approach to significantly advancing the performance of phase change thermal storage technology due to its broad-spectrum absorption capability, high carrier mobility, and abundant surface chemical activity.

This study summarizes the characteristic advantages of MXene/PCM for efficient and effective photovoltaic thermal storage. The advantages of composite thermal storage materials in light of the broad-spectrum absorption and efficient non-radiative relaxation of MXene have been analyzed, as they can synchronize solar energy capture and thermal energy storage, overcoming defects in the hysteresis of the photothermal responses of traditional PCMs. However, the oxidative degradation of MXene (with a refrigerated expiration date of only 3 to 6 months), the toxicity risk associated with scale-up preparation (due to the handling of fluorine-containing reagents), and the insufficient cyclic stability testing (indicated by a low number of photothermal cycles) remain significant bottlenecks for its practical application.

With the aid of rapidly advancing artificial intelligence and machine learning, the conformational relationship between the combination of MXene functional groups and thermal conductivity/photoabsorption can be explored to realize the rational design of materials. In the future, basic theoretical studies (e.g., phonon/iso-exciton coupling dynamics) should be deepened, while multidisciplinary cross-innovation should be promoted to unlock the full chain of application potential of MXene in the fields of energy and environment.

Some MXenes may exhibit cytotoxicity; therefore, the potential application domain should be carefully examined and analyzed in future work.

## Figures and Tables

**Table 1:** Comparison of photothermal materials.

Materials	Advantages	Disadvantages	References	Title 1	Title 2	Title 3
Noble metals (e.g., Au, Ag, Pt).	High-efficiency solar thermal conversion can be realized by LSPR.	The material has a narrow absorption band, which is expensive and oxidizes easily.	[6,12]	entry 1	data	data
Carbon-based material.	Wide spectral absorption.	Low launch rate.	[6]	entry 2	data	data
Semiconductor material.	Low cost, easy to synthesize, and not susceptible to photodegradation or bleaching.	Low concentration of free charge carriers and poor infrared absorption.	[6]			
Two-dimensional transition metal nitrides/carbides.	The bandgap is easy to adjust, and the in-plane electron mobility is high, which theoretically provides the most excellent photothermal conversion efficiency.	Poor long-term stability and toxicity of the synthesis process make it difficult to scale up.	[6,13,14]			

**Table 2:** Works of MXene-based phase change heat storage materials.

PCMs	Preparation Process	Changes in Thermal Parameters	Photothermal Capability	Research Gap	References
MgSO_4_·7H_2_O	The MXene colloidal solution was mixed with MgSO_4_·7H_2_O according to 1:1 and stirred and sonicated for 30 min.	During the 20 hydration/dehydration cycles, the heat release fluctuated less, and the thermal conductivity was 3.25 times higher than the original.	The average temperature of composite PCM was 15 °C higher than that of pure substance for the same light duration and intensity.	There is water vapor sorption and desorption in the storage heat cycle of hydrated salts, and the degradation of MXene under this condition needs to be considered.	[88]
Stearyl alcohol (SAL)	The feed ratio of SAL/MXene = 19:1 was placed in a vacuum chamber at 100 °C for 12 h at 0.08 MPa for melt adsorption.	The composite PCM did not leak throughout the heat charging process. The thermal conductivity improved from 0.353 W/(m·K) to 0.486 W/(m·K).	The average temperature of the composite PCM was 10 °C higher than that of the control at the same light duration and intensity.	The increase in MXene gravity can enhance thermal parameters, but there is a lack of discussion on the critical value of positive gains; for example, in combination with cost and the actual usage environment.	[89]
Paraffin Wax (PW)	PW and MXene were added to 20 mL of aqueous solution in different mass ratios and stirred for 3 h at 90 °C.	The thermal conductivity of composite PCM increases with increasing MXene loading up to 0.62 W/(m·K).	The temperature of the composite PCM with the highest loading was 38.25 °C higher than that of the lowest when the light duration and intensity were the same.	The paper points out that the latent heat and weight losses of the material are more severe after several phase change cycles and need to be further optimized.	[90]
n-eicosane (C_20_)	C_20_ and MXene were mixed in different mass ratios, stirred at 90 °C for 2 h, and uniformly coated on the upper and lower surfaces of the porous phase change layer.	After 200 phase change cycles, the latent heat of the composite PCM was reduced by only 2.58%.	Simulating sunlight irradiation, the maximum content of composite PCM reaches 89.07 °C, which is 22.82 °C higher than the minimum average temperature.	There is an ambivalence in the simultaneous improvement of thermal properties and mechanical strength of composites, and the choice of additive ratios should be discussed.	[91]
PEG	Different volumes of MXene aqueous dispersions were mixed with PEG and sonicated separately, and the precipitates were heated and melted at 80 °C.	The enthalpy remained almost constant after 200 thermal cycles.	Solar thermal conversion efficiency up to 90.45%.	Lack of a comparison with other composites in the same field.	[92]
Na_2_SO_4_·10H_2_O (SSD)	A certain mass of modified material and PCM were dissolved in MXene solution, irradiated with UV light at 50 °C for 2 h, and placed in an environment with 80% humidity for 24 h. The modified material and PCM were dissolved in MXene solution and irradiated with UV light at 50 °C for 2 h.	The phase change temperature and latent heat value remained almost unchanged after 50 cycles, with no obvious signs of leakage.	In the case of light irradiation, the composite PCM is able to absorb enough heat to complete the phase transition process, while the pure substance only increases in temperature and does not undergo a phase transition.	There is a lack of shape stability testing of the material after it has been subjected to multiple phase change cycles.	[93]
NaNO_3_	The aqueous phase was prepared by mixing and stirring 10 g of PCM, active agent with 5 mL of MXene aqueous dispersion, which was mixed with the oil phase and stirred thoroughly to form composite microcapsules.	The thermal conductivity of composite PCM was improved by 156.2%, the subcooling was reduced by 49.6%, and the thermal reliability of the composite PCM was 94% after 50 cycles.	Photothermal conversion efficiency increased by 169.4%.	The paper points out that the composites have a large degree of subcooling and need to be improved by the addition of nucleating agents to improve the deficiencies.	[94]
n-Octadecane (C_18_)	Trace amount of MXene was poured into the prepared emulsion and stirred at 800 rpm for 30 min. It was heated to 85 °C and then stirred for another 5 h and then dehydrated for 24 h. A sample was extracted from the sample and then dried.	Composite PCM showed the highest encapsulation rate, and the encapsulation process hardly affected the latent heat of the microcapsules, and the thermal conductivity increased by 52.3% compared with pure C_18_.	The solar thermal conversion efficiency reaches 85.7%, which is 240% higher than the undoped sample.	Some of the characterization experiments tested incomplete and insufficiently comparable materials.	[95]
D-mannitol (DM)	Different masses of DM were added to the MXene colloidal solution and stirred under nitrogen atmosphere for 48 h at room temperature and dried for 60 h to obtain the composite aerogel.	During continuous heating, aerogels with higher MXene content are less likely to leak and have faster heating and cooling rates, indicating better thermal conductivity.	Solar thermal conversion efficiency of up to 88.1%.	Lack of cycling performance tests for photothermal conversion.	[96]
PEG	A certain amount of MXene was added to 10 mL of PEG anhydrous ethanol solution of different concentrations and stirred at 80 °C for 30 min.	After 100 phase change cycles, the latent heat loss of the composite PCM was only 1%, and the acceleration of the charging and discharging process proved that the thermal conductivity was improved.	The higher the content of MXene, the higher the surface temperature of the composite PCM for the same irradiation time.	According to experimental data, sunlight radiation alone cannot raise the surface temperature of phase change materials to the melting temperature.	[97]
Myristic acid (MA)	Laser-processed MXene aerogel was immersed in the melted MA at 80 °C and vacuumed for 2 h	Composite PCM achieved 94.4% encapsulation with no leakage during the phase transition.	The solar energy absorption rate reaches 96% and the photothermal conversion efficiency reaches 93.5%.	Lack of cycling performance tests for photothermal conversion.	[98]
PEG	The MXene dispersion was mixed with PEG at a molar ratio of 1:2 and reacted for 1 h at 70 °C.	Composite PCM remains dimensionally stable during heating and is leak-free with higher thermal conductivity.	The thermal energy storage efficiency of composite PCM was 94.5% in the full solar spectrum.	Lack of a comparison with other composites in the same field.	[99]
PW	The T@G compound was immersed in the PW melt and dried and degassed for 30 min.	The thermal conductivity of composite PCM is 0.919 W/(m·K), which is 3.48 times higher than that of PW.	The sample with the most MXene doping had the highest temperature for the same light time and intensity.	Lack of cycling performance tests for photothermal conversion.	[100]

## Data Availability

No new data was created in this study.

## Abbreviations

The following abbreviations are used in this manuscript:

MXene	Two-dimensional transition metal carbides/nitrides
PCMs	Phase-change materials
IR	Infrared
UV	Ultraviolet
TES	Thermal energy storage
LSPR	Localized surface plasmon resonance
HF	Hydrofluoric acid
F-	Fluoride ions
OH-	Hydroxide ions
DMSO	Dimethyl sulfoxide
CVD	Chemical vapor deposition
STEM	Scanning transmission electron microscopy
EELS	Electron energy loss spectroscopy
NMP	N-methyl-2-pyrrolidone
DFT	Density functional theory simulations
SAT	Sodium acetate trihydrate
PEG	Polyethylene glycol
SAL	Stearyl alcohol
PW	Paraffin wax
C20	N-eicosane
SSD	Na2SO4·10H2O
C18	N-octadecane
DM	D-mannitol
MA	Myristic acid
